# Case Report: Reflection on a case of splenic abscess in a child

**DOI:** 10.3389/fped.2024.1407959

**Published:** 2024-09-11

**Authors:** Tao Zhou, Yongfang Liu

**Affiliations:** Department of Infectious Disease Department, The Third People’s Hospital of Chengdu, Chengdu, China

**Keywords:** splenic abscess, child, aseptic splenic abscess, pathogenic, diagnostics, treatment

## Abstract

Splenic abscesses (SA), especially in children, are rare in clinical practice. The missed diagnosis rate of SA is high and the probability of rapidly diagnosing it is relatively low due to its low incidence rate and the presence of non-specific clinical symptoms and imaging manifestations. Antibiotics are the primary treatment for SA; however, ultrasound-guided percutaneous puncture suction or drainage, and splenectomy are other effective treatment strategies. In this study, we report one case of SA in a 16-year-old male patient who presented with abdominal pain, fever, and cough, and the therapeutic effect was unsatisfactory (recurrent fever). After admission, the patient was diagnosed with a solitary SA by abdominal CT with contrast and upper abdominal MRI; fever and abdominal pain were relieved and the SA gradually disappeared after antibiotic treatment.

## 1 Background

Splenic abscesses (SA), especially in children, are rare in clinical practice, with previous studies based on autopsy cases revealing an incidence rate: 0.14%–0.7% (1, 2). SA generally occurs in males and individuals with immunodeficiency (3, 4), manifesting as fever and abdominal pain. Due to no specific clinical manifestations, the diagnosis of SA is easily overlooked, leading to a poor prognosis and a high mortality rate. In this study, we report the diagnosis and treatment of a case of SA in a child. Here, we aimed to enhance the vigilance of splenic abscess and reduce missed diagnosis and misdiagnosis rates.

## 2 Case study

A 16-year-old male patient was admitted due to abdominal pain for more than one month and intermittent fever for two weeks. More than one month before admission, the patient developed middle upper abdominal pain after eating, which lasted for about 7–8 h and then self-relieved, accompanied by nausea. The patient received examinations (examination items are unknown) in another hospital, and the abdominal pain was alleviated after treatment (medications are unknown). However, during the disease, he became febrile with a maximal temperature of 39℃, accompanied by cough, expectoration, dizziness, headache, tightness of breath, joint pain in both hands, and weakness in the limbs. Pathology assessment for Mycoplasma DNA, SARS-CoV-2, and influenza A and B antigens were negative, and no significant abnormality was found on chest computed tomography (CT). Routine blood tests (November 23rd, 2023) showed the white blood cell (WBC) count was 13.82*10^9 ^/L, the neutrophil count was 10.85*10^9^ /L, the percentage of neutrophils was 78.5%, and the C-reactive protein (CRP) level was 18.49 mg/L. The patient was administered cefuroxime (1.5 g twice a day for 3 days by intravenous infusion, 0.5 g twice a day or 3 days by oral administration) and symptomatic treatment. The patient's WBC returned to normal; however, the patient had a recurrent fever, with body temperature fluctuating between 37.5℃ and 37.8℃. Two days before admission, the patient developed a more severe dry cough. Since the onset of the illness, the patient had lost 2–3 kg of weight. Additionally, he also gave a history of intermittent diarrhea for more than 10 years with no prior relevant investigations. Following administration, the patient reported an allergy to cefixime and subsequently developed a rash on the lower limbs. The patient denied any history of allergies to penicillin, sulfonamide drugs, streptomycin, or food. The patient's vaccination history was unknown. In October 2022, the patient underwent surgical treatment due to appendicitis in our hospital. The patient had no history of other underlying diseases, infectious diseases, trauma or blood transfusion, and there was no remarkable personal or family history.

Abdominal examination revealed distension, tenderness and rebound tenderness at the left upper quadrant, as well as tenderness and rebound tenderness at McBurney's point, without any other notable positive signs. After admission, the patient actively received relevant auxiliary examinations. Routine blood tests showed that the WBC was 9.81*10^9^ /L, the N count was 6.61*10^9^ /L, the N% was 67.4%, the CRP was 5.81 mg/L, and the erythrocyte sedimentation rate (ESR) was 26 mm/h. Other laboratory indicators were within the normal range, including procalcitonin, interleukin-6, blood biochemical examination, cardiac markers, lipase, amylase, coagulation test, thyroid function, connective tissue disease-related antibodies, γ-interferon release experiment, Widal and Weil-Felix tests, and the D-glucan test. Pathogenic examination revealed no HIV, syphilis, tuberculosis, mycoplasma pneumoniae, or viruses (e.g., cytomegalovirus, herpes simplex, SARS-CoV-2, rubella virus, influenza A virus, influenza B virus, respiratory syncytial virus, adenoviruses, human rhinovirus). Additionally, sputum culture and blood cultures were all negative. Chest CT showed small inflammatory nodules in the right middle lobe and left lower lobe of the lung. Abdominal CT with contrast showed a low-density lesion in the spleen, appearing as a weak enhancement at the edge of enhanced scanning, leading to the consideration of abscess or alternative pathologies; diagnosis was to be established on clinical manifestations and MRI examinations. Cardiac ultrasound, electrocardiogram and colonoscopy examination showed no notable abnormalities.

**Figure 1:**
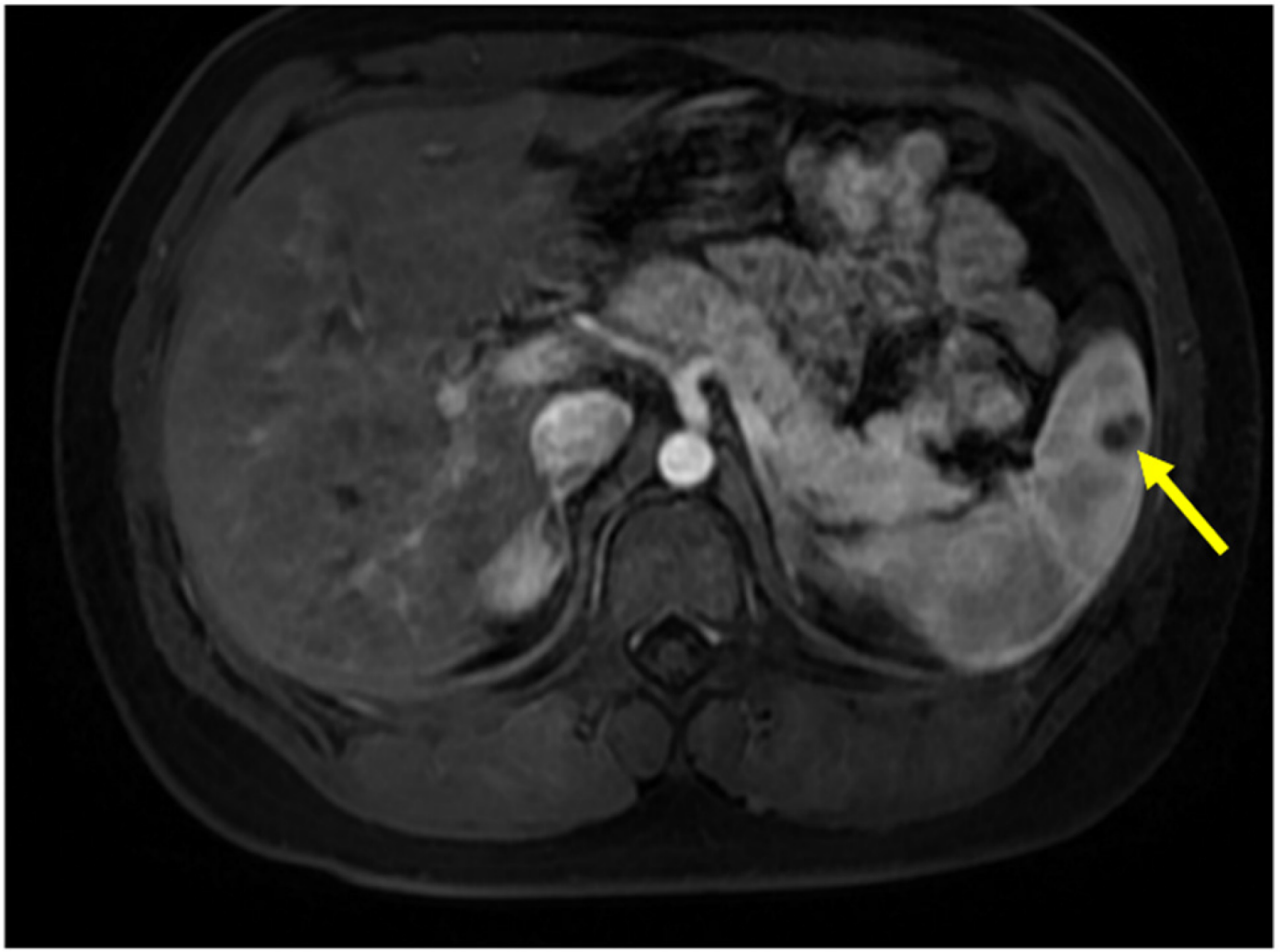
Contrast-enhanced MRI showing a low-density lesion in the spleen (approximately 13 mm).

**Figure 2:**
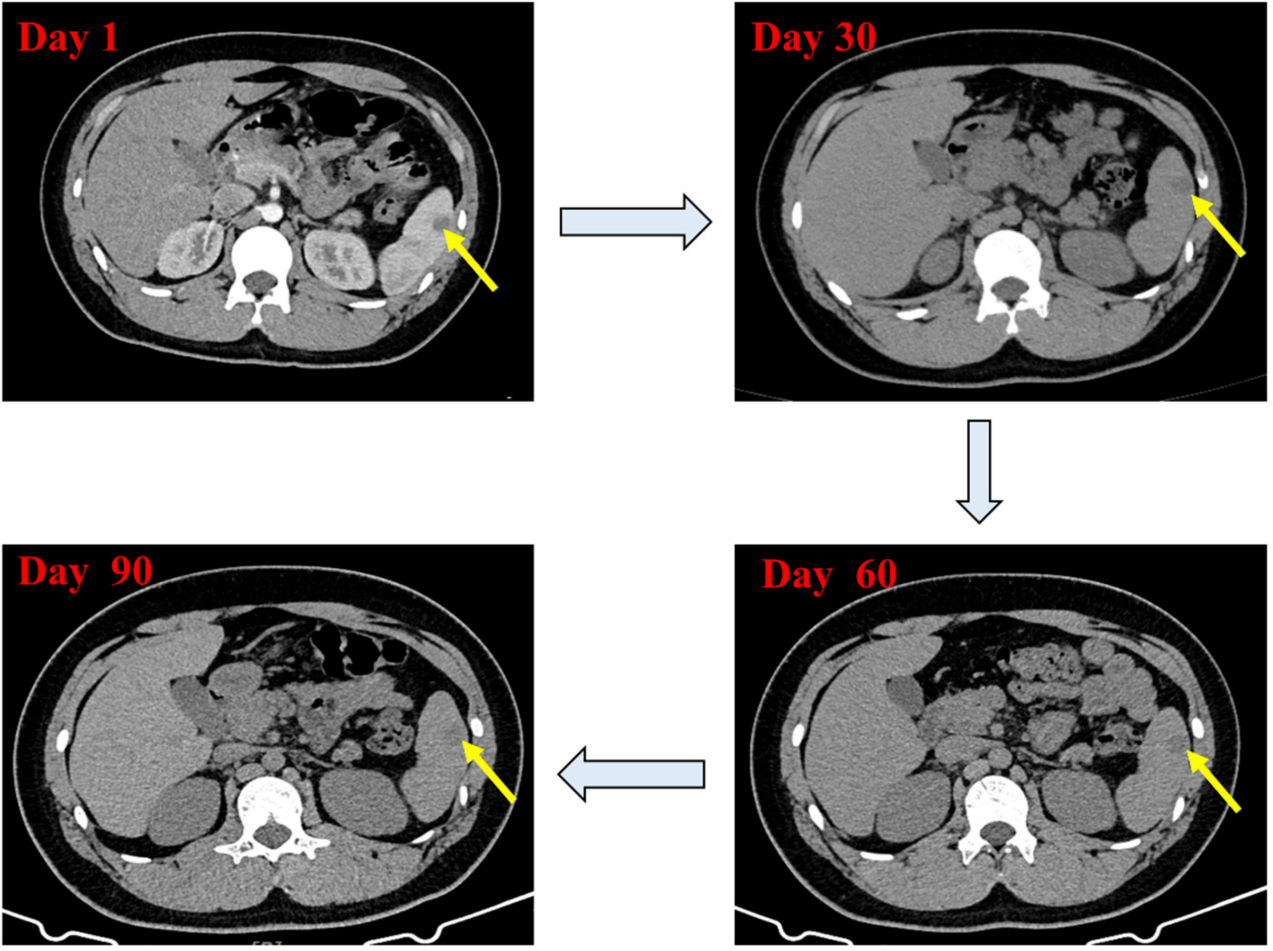
Contrast-enhanced abdominal CT scan showing changes in the low-density lesion in the spleen during treatment.

Based on the results of abdominal CT with contrast, The diagnosis of splenic abscess needs to be considered. Due to the lack of pathogenic evidence, empirical anti-infection therapy with piperacillin-tazobactam (4.5 g every 8 h) was administered. The patient underwent upper abdominal MRI plain scanning and contrast-enhanced imaging to further elucidate the SA. The results showed: abnormal nodules in the spleen, inclined towards benign lesions. In addition to anti-infection treatment, the hepatobiliary surgery department was consulted for assistance in diagnosis and treatment. The consultation recommended: (1) Conservative treatment, with follow-up after anti-infection treatment; (2) Ultrasound-guided puncture biopsy/drainage; (3) Surgical procedures. The patient had a small abscess (approximately 13 mm, as shown in Figure 1). After communicating with physicians in the ultrasound department experienced in puncture and weighing the pros and cons of the surgical risks, the patient's family finally chose to continue with medication for anti-infection treatment. During hospitalization, the patient's abdominal signs gradually improved and no fever occurred. The inflammatory indicators returned to normal after one week of anti-infection treatment. Therefore, anti-infection treatment was continued and follow-up was conducted after discharge (once a month). Finally, after taking antibiotic for 3 months, the patient's general condition was normal, and the imaging lesions became lighter and smaller than upon admission. Antibiotics were discontinued, and the patient was followed up again at the outpatient clinic after one month of without medication. The patient had no abdominal pain, and the general condition and blood routine examination was normal. Abdominal CT scans were performed after one, two, and three months of treatment, as shown in Figure 2. During hospitalization and outpatient follow-up, the patient's temperature, degree of abdominal pain, white blood cell count, and CRP level were re-examined, as shown in Table 1.

**Table 1:** The patient's temperature, degree of abdominal pain, white blood cell count, and C-reactive protein level during treatment.

Time of treatment	Temperature (°C)	Abdominal pain	The count of white blood cell (Reference range of normal value: 4.1–11*10^9^ /L)	C-reactive protein (mg/L, Reference range of normal value <10 mg/L)
Day 1	37.3℃	Obvious	9.81*10^9 ^/L	5.81 mg/L
Day 7	36.8℃	Slight	7.41*10^9 ^/L	2.72 mg/L
Day 30	36.5℃	Slight	6.35*10^9 ^/L	3.09 mg/L
Day 60	36.3℃	No	5.84*10^9 ^/L	4.51 mg/L
Day 90	36.5℃	No	5.72*10^9 ^/L	4.84 mg/L

## 3 Discussion

SA in adults is often accompanied by combined immunodeficiency diseases, application of immunosuppressants (5), diabetes (6), infectious endocarditis (7), splenic infarction (7), splenectomy, and other immune dysfunction, malignant or infectious diseases, hematological diseases, and ventricular assist device implantation (8). SA may also occur after tooth extraction due to septic embolism (9). The incidence of SA in children with underlying diseases is significantly lower than that observed in adults, and notably, trauma and diabetes are uncommon in pediatric cases. Hence, significant distinctions exist between splenic abscess presentations in children and adults: First, the age of onset of SA in children is usually over 10 years old and is more common in males than females; Second, bloodstream infections, hematological diseases, typhoid fever, EB virus infections, malaria and tuberculosis commonly cause SA in children. Infectious endocarditis, tympanitis and appendicitis are common causes of secondary infections. Among SAs caused by hematological diseases, leukemia is the most common, followed by aplastic anemia, and anemia caused by other reasons (10, 11).

Interestingly, not all SA cases have an infectious etiology. Indeed, SA cases can be divided into infectious and aseptic. The most common pathogenic bacteria for infectious SAs are streptococcus and *Escherichia coli* (12, 13), gram-positive cocci and anaerobic bacteria. Additionally, there are reports of SAs (14, 15) caused by *Clostridioides difficile* (16), *Mycobacterium tuberculosis* (17) and *Salmonella typhi* (18). There have also been reports of fungal infections causing SAs in populations with hypoimmunity (19, 20). Meanwhile, it has been reported that brucella (21), burkholderia pseudomallei (22), and hydatid (23) could cause splenic abscesses. In contrast, aseptic SA is usually a manifestation of other diseases, including Sweet syndrome (24), systemic lupus erythematosus (25), and inflammatory bowel diseases [e.g., Crohn's disease (26), ulcerative colitis (27), Behçet's disease (28), and pyoderma gangrenosum (29)]. The treatment of these diseases generally requires corticosteroids or other immunosuppressive agents.

There is no recognized and definite treatment for pediatric SA. Anti-infection treatment is the basic choice for infectious SA. If not improved, abscess drainage or splenectomy may be another choice. The antibiotic treatment dose and duration are adjusted based on blood culture sensitivity results. For SA smaller than 3 cm, antibiotic treatment is mainly administered intravenously in sufficient amounts, with an indefinite course; however, depending on the severity of the infection, the course should last for at least 4 weeks. Some patients, despite receiving sufficient antibiotic treatment, still have limited improvement, and percutaneous ultrasound-guided abscess drainage or even splenectomy is required (30). Moreover, dynamic follow-up on ultrasound or abdominal CT is required during the treatment. It is necessary to evaluate whether percutaneous puncture and suction or drainage is required in patients with the diameter of the abscess greater than 3 cm.

## 4 Conclusions

In the current case, the patient's abscess was small, allowing intravenous treatment with a single antibiotic. Unfortunately, we could not obtain clear pathogenic evidence in this case due to the lack of puncture drainage, and negative blood culture. Therefore, we empirically administered intravenous anti-infection treatment with piperacillin-tazobactam. The patient did not develop a fever during the treatment, and the abdominal signs improved significantly, suggesting that the antibiotic was effective. Thus, we excluded the possibility of abscesses caused by tuberculosis, fungi, malaria, other pathogens, and immune diseases.

## Data Availability

The original contributions presented in the study are included in the article/Supplementary Material, further inquiries can be directed to the corresponding author.
